# Wearing a Surgical Face Mask Has Minimal Effect on Performance and Physiological Measures during High-Intensity Exercise in Youth Ice-Hockey Players: A Randomized Cross-Over Trial

**DOI:** 10.3390/ijerph182010766

**Published:** 2021-10-14

**Authors:** Keely A. Shaw, Scotty Butcher, Jong Bum Ko, Abdi Absher, Julianne Gordon, Cody Tkachuk, Gordon A. Zello, Philip D. Chilibeck

**Affiliations:** 1 College of Kinesiology, University of Saskatchewan, Saskatoon, SK S7N 5B2, Canada; keely.shaw@usask.ca (K.A.S.); jongbum.ko@usask.ca (J.B.K.); asa727@mail.usask.ca (A.A.); julianne.rooke@usask.ca (J.G.); 2 School of Rehabilitation Science, University of Saskatchewan, Saskatoon, SK S7N 5B2, Canada; Scotty.butcher@usask.ca; 3 Pediatric Physical Therapy, Jim Pattison Children’s Hospital, Saskatoon, SK S7N 5B2, Canada; cnt628@mail.usask.ca; 4 College of Pharmacy and Nutrition, University of Saskatchewan, Saskatoon, SK S7N 5B2, Canada; gordon.zello@usask.ca

**Keywords:** physical activity, coronavirus, pulse oximetry, near-infrared spectroscopy, COVID-19, pandemic, performance, surgical mask

## Abstract

COVID-19 transmission is prevalent during ice-hockey; however, it is unknown whether wearing face masks as a mitigation strategy affects hockey players’ performance. We used a randomized cross-over study to compare wearing a surgical mask to a sham mask (control) in youth hockey players (21 males, 5 females, 11.7 ± 1.6 y) during a simulated hockey period (cycle ergometry; six shifts of 20 s of “easy” pedaling (40% peak power), 10 s of “hard” pedaling (95% peak power), 20 s of “easy” pedaling, with shifts separated by 5 min rests). A seventh shift involved two 20 s Wingate tests separated by 40 s rest. Heart rate, arterial oxygen saturation and vastus lateralis tissue oxygenation index (hemoglobin saturation/desaturation) was assessed each shift. On-ice testing was conducted with the maximal Yo-Yo intermittent recovery test. No differences between mask and control conditions for performance were found (Wingate average power: 245 ± 93 vs. 237 ± 93 W, Peak power: 314 ± 116 vs. 304 ± 115 W, on-ice distance: 274 ± 116 vs. 274 ± 110 m) and for heart rate or arterial oxygen saturation during simulated hockey shifts. Tissue oxygenation index was lower from shifts one to six for males (*p* < 0.05) and shift seven for females (*p* < 0.01) while wearing a mask. Wearing a face mask had no effect on performance in hockey players with only minor effects on muscle oxygenation. ClinicalTrials.gov (NCT04874766) (accessed on 6 May 2021).

## 1. Introduction

The COVID-19 virus is spread through direct and indirect means from an infected individual [1]. Sporting events such as ice hockey pose a high level of risk for transmission due to the close proximity of players, playing in enclosed spaces with poor ventilation and heavy breathing that can be expected during this type of exercise [2]. Observations from infections within hockey teams have described transmission from a single asymptomatic carrier leading to the quarantine of as many as six different teams in the league and multiple infections [2,3]. Children or adolescents who are infected during sports such as hockey have the possibility of transmitting COVID-19 to more vulnerable family members [4,5].

Due to the COVID-19 pandemic, many youth sport leagues were suspended for weeks or months at a time in 2020 and 2021, leading to reduced participation across millions of young athletes [6]. Given the importance of sport in promoting positive health, social, emotional, cultural and economic outcomes, this reduction in sport participation could potentially lead to long-term negative consequences for those impacted directly as well as for the community at large [7]. With the cancellation of various sport leagues, children are missing the opportunity to develop physical literacy, engage in physical activity and develop values that sport provides [8]. Given the increase of COVID-19 variants of concern [9] and the inability of children under the age of 12 to be vaccinated at the time of writing, it is important to investigate possible ways to allow youth to continue to engage in sport during the COVID-19 pandemic while minimizing the risk of viral spread. 

The use of face masks in public areas is one way to limit the spread of infectious respiratory droplets and, thus, COVID-19 transmission [10]. However, some apprehension has been identified around wearing face masks during exercise for concerns of inadequate oxygenation and an increase in carbon dioxide rebreathing [11]. We recently conducted a systematic review that reports wearing a face mask during exercise is not only safe but has minimal impact on physiological or performance variables across a variety of clinical and healthy populations [12]; however, there is a recent study that indicates a negative effect on performance, physiological measures (i.e., maximal oxygen consumption) and increased dyspnoea when wearing face masks during exercise [13]. To our knowledge, only a single study has been reported in the use of face masks in children and youth during physical activity [14]. During “mild exertion” (i.e., brisk walking), a small increase in the fraction of inspired carbon dioxide while wearing a face mask was found, but with no differences compared to not wearing a facemask for other physiological measures (i.e., heart rate, respiratory rate and arterial oxygen saturation). We are unaware of any research investigating the physiological effects of wearing a face mask during an intense team sport such as ice hockey. 

The purpose of the current research was to assess the impact of wearing a face mask during a simulated hockey period and a progressive skating test in youth hockey players aged 9–14 years. Given the previous work from our lab [12,15], which suggests that individuals of various ages and health statuses can wear a mask during exercise with no impacts on performance, we hypothesized that physiological and performance variables of youth hockey players would be unaffected while wearing a face mask during a simulated hockey period and progressive-intensity skating test.

## 2. Materials and Methods

**Figure 1:**
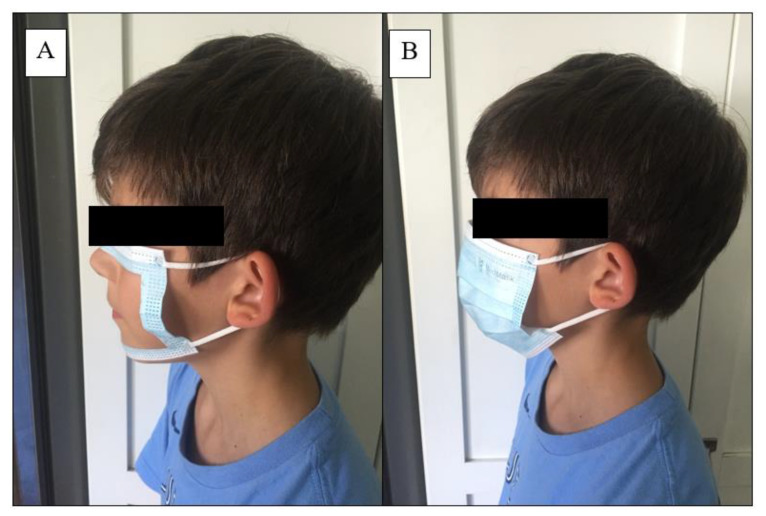
Fitting of the sham (**A**) and regular (**B**) masks (photo use by permission).

This study utilized a randomized double-blind counterbalanced cross-over design in which 26 youth hockey players (21 males, 5 females; age 11.7 ± 1.6 y) were randomized to wear either a three-layer surgical face mask (AMD Medicom Inc, Quebec, QC, Canada) or a “sham” mask, which had a hole cut out of it to expose the mouth and nose (as described by Mapelli et al. [16] (Figure 1A,B)) while completing a simulated hockey period on a cycle ergometer, as well as during an on-ice skating performance assessment. Participants then crossed over to the other condition (i.e., face mask or sham mask) for identical testing on a different day. Three-ply disposable surgical masks were chosen as they are readily available to the general public and are effective for reducing the transmission of exhaled droplets [17]. “Double-blinding” was for the participant (i.e., participants were only told that different masks were being assessed) and the individual analyzing data (these conditions are coded as “A” and “B”). All personnel who were involved in testing participants and collecting data could not be blinded to conditions. All sessions were separated by at least 24 h. Participants were stratified by sex and then randomized using block sizes of four using a random number generator. The researcher who generated the allocation schedule concealed the schedule from the researchers who were enrolling participants.

Participants were invited to take part in the study if they were between 9 and 14 years of age and had played organized hockey during the previous season (2020/2021). Participants were recruited through word of mouth and through contacting local hockey coaches. This research was approved by the University of Saskatchewan Research Ethics Review Board (Ethics approval #Bio 2701). Parents of participants consented to the study and participants provided assent. The study was registered at clinicaltrials.gov (NCT04874766) prior to participant recruitment. 

Sample size was based on previous findings that performing repeated Wingate tests in mild hypoxic conditions (i.e., in environments with reduce atmospheric partial pressure of oxygen) results in an approximate 3% decrement in average power during repeated Wingate tests [18]. For the ages of our children, average power during Wingate tests is about 450 W with a standard deviation of 40 W [19,20]. A 3% reduction would result in an average power of 436 W. Assuming a correlation of 0.9 between repeated measures and using an alpha of 0.05 and power of 90%, this resulted in a required sample size of 20 (Statistica 7.0, Statsoft, Chicago, IL, USA). 

### 2.1. Off-Ice Tests

Off-ice testing involved a simulated hockey period consisting of seven shifts performed on a cycle ergometer and occurred over 4 different visits to the lab. The first session involved a modified 20 s Wingate anaerobic test without a mask, which has previously been deemed to be appropriate for children [20]. After a 2 min warm-up involving pedaling at 60 rpm against 0.075 kg per kg of body mass and a one-minute passive rest period, participants pedaled maximally against 0.075 kg per kg of body mass for 20 s [18,19]. Peak and average power output were recorded. Data from this session were used to set the workloads for all subsequent off-ice sessions.

Visits 2 to 4 to the lab all involved a simulated hockey period performed on a cycle ergometer. Cycle ergometry closely approximates the metabolic stress induced in the quadriceps muscles (i.e., the muscle from which we measured tissue oxygenation index; see below) during ice-hockey [21] and is, therefore, often used as an off-ice testing modality for hockey players [19,22]. Session two was completed with no mask and served as a familiarization trial to decrease any learning effects and confirm the appropriateness of the selected power outputs for individual participants. During session three, the participants wore either a surgical mask or a sham mask and on session four wore the other mask condition.

### 2.2. Simulated Hockey Period

A typical hockey game involves working at hard intensities for about 18% of the time and low intensities for about 82% of the time, with shifts separated by bench rests of ~five minutes [23]. Approximately seven shifts occur per 20 min period in hockey [23,24,25]. These demands were replicated on a cycle ergometer (Lode Corival cpet, Groningen, The Netherlands). This bike was chosen because it maintains a stable power output, regardless of the cadence of the participants. 

**Figure 2:**
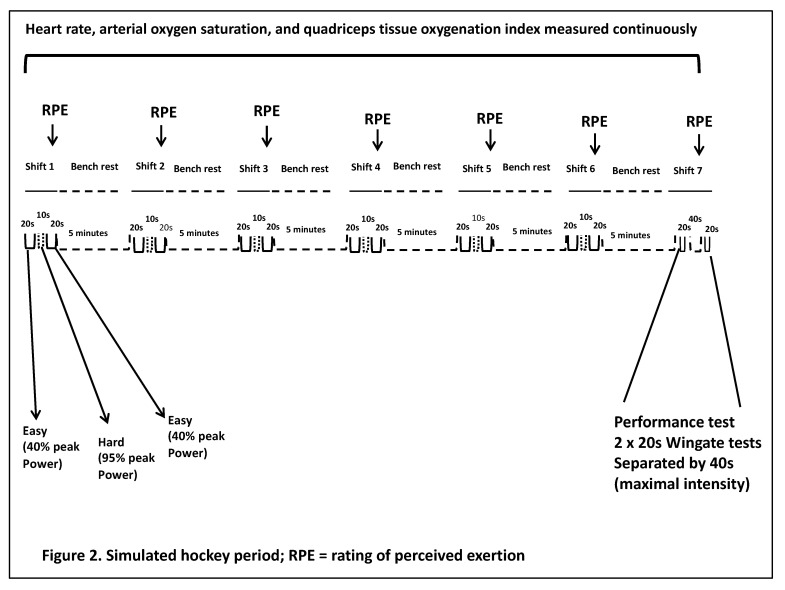
Simulated hockey period; RPE = rating of perceived exertion; HR = heart rate; SpO_2_ = atrial oxygen saturation.

Participants completed six “shifts”, which included 20 s at 40% peak power output from session 1, 10 s at 95% peak power output, followed by another 20 s at 40% peak power output. Each shift was followed by a five-minute “bench rest”, where they sat passively. The seventh “shift” involved two 20 s modified Wingate anaerobic tests (as was completed in session one) separated by 40 s of passive rest. This intensified seventh “shift” was chosen to simulate an increased intensity that might be experienced at the end of a hockey period or game. Our primary dependent variable was average power output from these Wingate tests. A secondary variable was peak power output. A schematic representation of the simulated hockey period is shown in Figure 2.

During sessions three and four, heart rate and arterial blood oxygen saturation were monitored with a pulse oximeter (Nellcor Oximax NPB40MAX, Medtronic Canada, Brampton, ON, Canada) and a rating of perceived exertion (Modified Borg scale; i.e., 10-point scale) was recorded at the end of every “hard” (95% peak power output) phase, as well as at the end of the repeated Wingate tests. Continuous-wave near-infrared spectroscopy (NIRS; NIRO-200NX, Hamamatsu Photonics, Hamamatsu City, Shizuoka Pref., Japan) was measured continuously and used to measure the tissue oxygenation index of the right vastus lateralis, as previously described [15].

### 2.3. On-Ice Testing

Sessions five and six involved on-ice testing (Granatier Sportsplex, Saskatoon, SK, Canada), completed in full equipment with stick in hand. Participants were randomized to wear either a mask or a sham mask (as described above) for session five and the other condition for session six. Prior to testing, participants completed a standardized warmup, where they were instructed to complete two laps of the 95 × 50 ft hockey rink at an “easy” pace, one lap at a “hard” pace and to finish with two easy laps. Participants then completed the maximal Yo-Yo Intermittent Recovery test (level 1) [26]. In short, participants skated between two cones spaced 20 m apart following the sounds of an audio recording (https://www.youtube.com/watch?v=NPuPJ_7WIBI&t=337s, accessed on 20 June 2021), with the sounds getting closer together with each 20 m repeat, such that required skating speed increased progressively. Each run between the cones was separated by a 10 s recovery period. Participants were given one warning if they did not reach the start/finish cone in time. The test was terminated the second time the participants did not reach the start/finish cone in time. Participants were given standardized verbal encouragement to continue to exhaustion. Total distance covered, as well as final level (i.e., velocity), was recorded. 

To control for the effects of diet, previous physical activity and sleep, participants were required to record their physical activity, dietary intake and sleep in the 24 h prior to session three and replicate them before session four. Similarly, participants were required to record their physical activity, dietary intake and sleep prior to session five and replicate prior to session six. 

### 2.4. Statistical Analyses

Data are presented as means ± SD. Data were assessed for normality using the Shapiro–Wilk test. Statistical analyses were completed using Statistica 7.0 software (Statsoft, Chicago IL, USA). On-ice variables (i.e., distance travelled and final speed) were assessed using a sex (male vs. female) × mask (mask vs. sham) repeated-measures ANOVA with repeated measures on the “mask” factor. Heart rate, ratings of perceived exertion, arterial oxygenation, muscle oxygenation and peak and average power during the Wingate tests were assessed using a three-way (sex × mask × time) repeated-measures ANOVA, with repeated measures on the “mask” and “time” factors. Least-square difference (LSD) post hoc tests were used to compare differences if significant main effects or interactions were observed. Group means for a given time point were imputed for any missing values. Our main analysis was an “intent-to-treat” analysis with missing values filled in. We also conducted secondary analyses where participants with missing values were excluded. Significance was accepted at *p* ≤ 0.05.

## 3. Results

### 3.1. Exercise Performance

**Figure 3:**
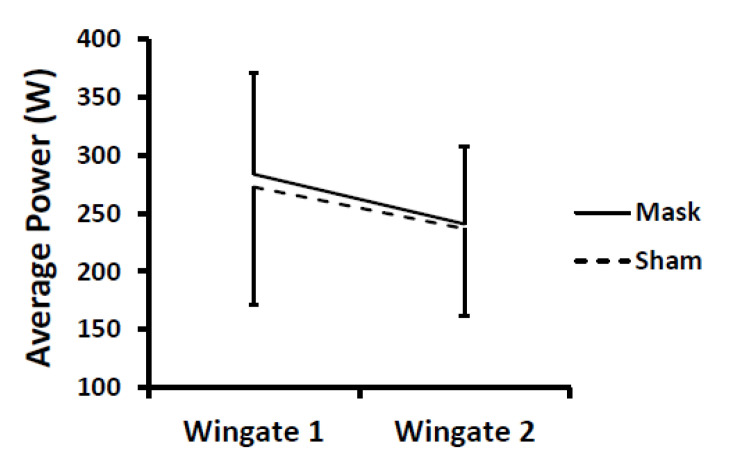
Change in average power during repeated Wingate tests for mask and sham conditions. There were no differences between conditions. There was a time main effect (*p* < 0.001) with Wingate 2 < Wingate 1. Values are means and error bars are SD.

**Figure 4:**
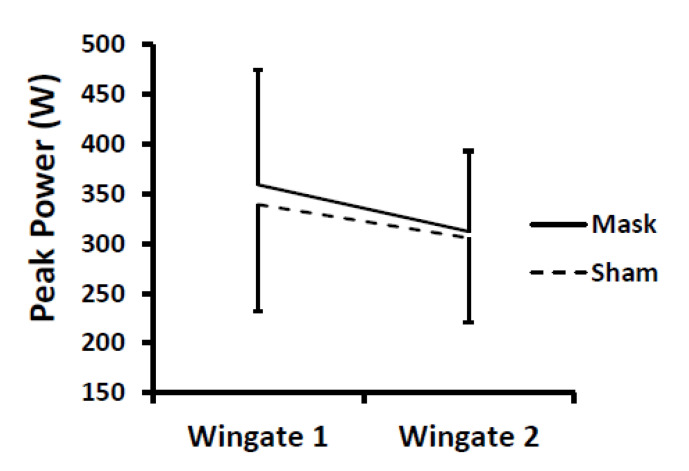
Change in peak power during repeated Wingate tests for mask and sham conditions. There were no differences between conditions. There was a time main effect (*p* < 0.001) with Wingate 2 < Wingate 1. Values are means and error bars are SD.

All participants completed the testing with no dropouts. After Wingate testing, one female participant vomited after the familiarization session; therefore, she only performed one Wingate test at the end of each simulated period during the mask and sham conditions. One male participant vomited while cooling down after the Wingate tests during the mask condition but had no issues during the sham condition. Peak and average power data were missing for a third participant (male) for the second Wingate during the sham condition due to a technical error. Group means were used to fill in these missing values. For average power and peak power during the Wingate tests, no differences were found between mask and sham conditions (i.e., no mask main effect and no mask × time or sex × mask × time interactions; *p*-values = 0.38–0.95) (Figure 3 and Figure 4). There was a time main effect (*p* < 0.001), as expected, with the second Wingate test lower than the first Wingate test. When the two participants with missing data were excluded from analyses and only participants with complete data were analyzed, the statistical results did not differ (i.e., no mask main effect and no mask × time or sex × mask × time interactions; *p*-values = 0.46–0.73). The work rate for each shift is displayed in Table 1.

All values are means ± standard deviation. The work rates for shifts 1–6 were fixed, based on peak power output achieved during baseline Wingate tests (40% and 95% peak power output for easy and hard intervals, respectively). 

**Figure 5:**
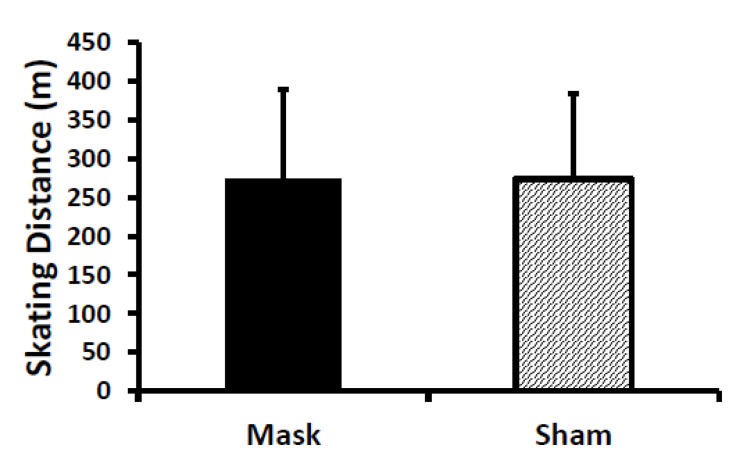
Skating distance during the maximal Yo-Yo Intermittent Recovery on-ice test. There was no difference between the mask and sham conditions. Values are means and error bars are SD.

For on-ice testing, no difference was evident between conditions (i.e., no mask main effect and no sex × mask interaction) for distance covered during the maximal Yo-Yo Intermittent Recovery test (Figure 5). Likewise, final velocity during the test was similar between conditions (Mask: 12.3 ± 1.1 km/h; Sham: 12.3 ± 1.1 km/h; *p* = 0.67).

### 3.2. Physiological Measurements

**Figure 6:**
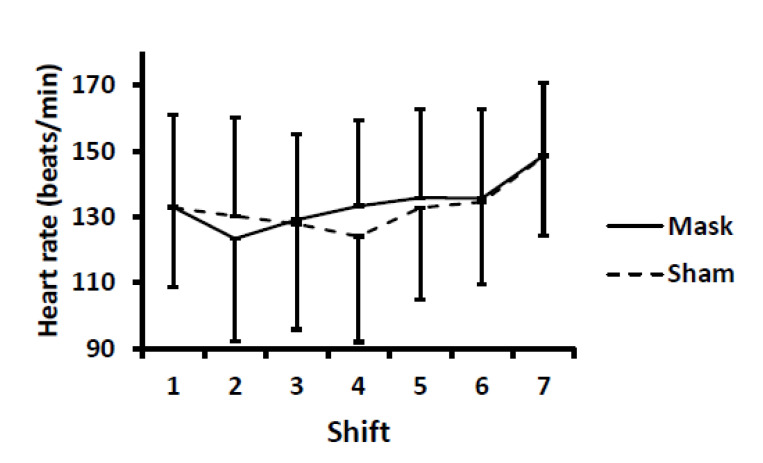
Heart rate during each simulated hockey shift. There were no differences between mask and sham conditions. There was a time main effect (*p* < 0.01) with heart rate higher after the seventh shift (i.e., the repeated Wingate tests) compared to all other shifts. Values are means and error bars are SD.

**Figure 7:**
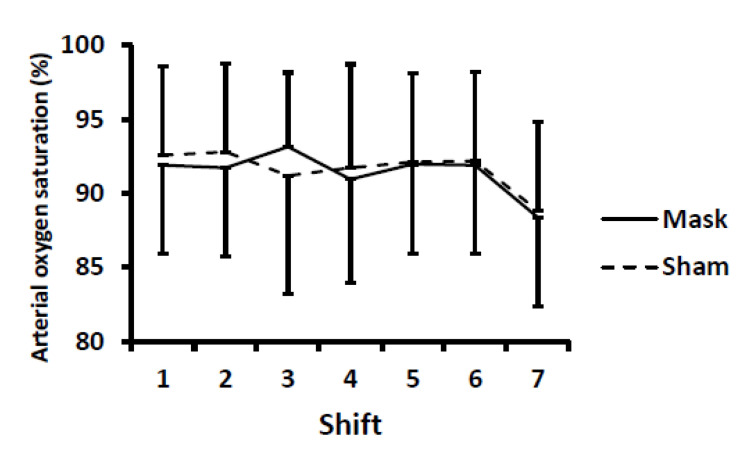
Arterial oxygen saturation during each simulated hockey shift. There were no differences between mask and sham conditions. There was a time main effect (*p* < 0.01) with oxygen saturation lower after the seventh shift (i.e., the repeated Wingate tests) compared to all other shifts. Values are means and error bars are SD.

For heart rate and arterial oxygen saturation, no differences were evident between conditions (i.e., no mask main effect and no mask × time or sex × mask × time interactions) (Figure 6 and Figure 7). There were time main effects, with heart rate higher and arterial oxygenation lower (*p* < 0.01) after shift seven (i.e., after the Wingate tests) compared to all other shifts. Due to equipment malfunction, 9 and 11 data points (out of 364 possible data points) were not recorded for arterial oxygenation and heart rate, respectively. Statistical results did not differ whether average values were imputed for missing data points or when those with missing data were excluded from the analyses.

**Figure 8:**
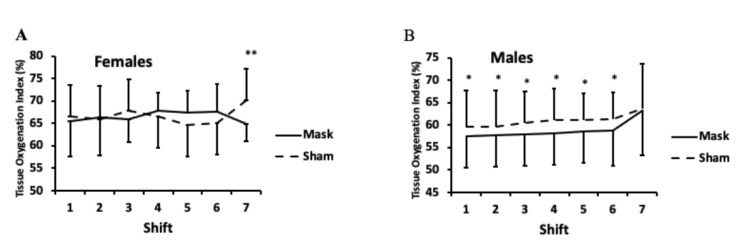
Tissue oxygenation index for females (**A**) and males (**B**). There was a sex × mask × time interaction (*p* < 0.01). LSD post hoc testing: ** Tissue oxygenation index was lower during the mask compared to the sham condition after shift 7 for females (*p* < 0.01). * Tissue oxygenation index was lower during the mask compared to the sham condition during shifts 1–6 for males (*p* < 0.05). Values are means and error bars are SD.

For the tissue oxygenation index, one male participant was excluded because of equipment failure during one of their conditions. For the remaining participants (*n* = 25), there was a sex × mask × time interaction (*p* < 0.01). For females, tissue oxygenation index was lower during the mask compared to sham condition after the seventh shift (i.e., the Wingate tests; *p* < 0.01; Figure 8A). Yet, for males, there was a lower tissue oxygenation index during the first six shifts for the mask compared to the sham condition (*p* < 0.05), but no difference after the seventh shift (Figure 8B). Four data points (out of 350 possible data points) were not recorded because of equipment malfunction. Statistical results did not differ whether average values were imputed for missing data points or when those with missing data were excluded from the analyses.

**Figure 9:**
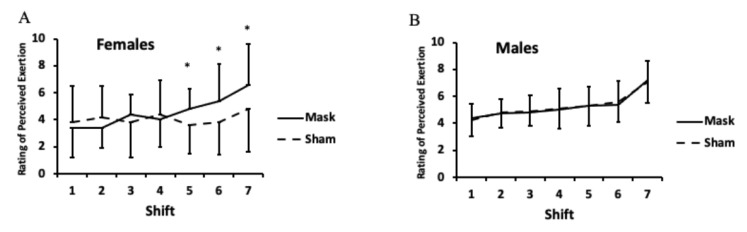
Rating of perceived exertion for females (**A**) and males (**B**). There was a sex × mask × time interaction (*p* < 0.01). LSD post hoc testing: * Rating of perceived exertion was higher during the mask compared to the sham condition during shifts 5–7 for females (*p* < 0.05). There were no differences between mask and sham conditions for males. Values are means and error bars are SD.

For rating of perceived exertion, a sex × mask × time interaction was apparent (*p* < 0.01). For females, there were higher ratings of perceived exertion for the mask versus sham condition during shifts 5–7 (*p* < 0.05; Figure 9A); whereas for males no differences between conditions at any time point were found (Figure 9B).

## 4. Discussion

Our most important finding was that wearing a face mask during a simulated hockey period had no influence on heart rate, arterial oxygen saturation, or Wingate performance. On-ice skating performance was also unaffected. For other variables measured, some minor significant differences were evident. Ratings of perceived exertion were not impacted for males, however, females reported increased ratings of perceived exertion during shifts five, six and seven in the masked versus sham conditions. Males had lower tissue oxygenation index at the vastus lateralis when wearing a mask during shifts 1–6 but no difference during the seventh shift, while females had no difference between conditions for the first six shifts but had decreased oxygenation in the masked versus sham condition for the seventh shift. Given that the potential for viral spread is high in a hockey environment [2,3], our findings are important not only during the COVID-19 pandemic but also beyond, as common viruses such as the common cold or influenzas also spread through respiratory droplets and cases tend to increase in the fall and winter months when children are often playing hockey.

The results of the study are consistent with our research hypothesis that wearing a mask would have no impact on exercise performance in youth hockey players. However, we did find that tissue oxygenation in the quadriceps was lower in the masked versus sham condition for the submaximal “shifts” in males and the maximal “shift” in females. While these decreases in muscle tissue oxygenation were statistically lower in the masked vs. sham condition, the absolute difference between masked and unmasked conditions was quite small (0.4–2.8%) and within the range observed in previous research in adults during masked and unmasked exercise [15], as well as in research involving children of a similar age during exercise without wearing masks [27]. Thus, any differences are likely to have minimal clinical importance. The observed sex differences in the current research suggest that females may be more efficient at submaximal intensities relative to males. This is supported by our previous research with face masks during exercise [15], where we also observed higher tissue oxygenation in females relative to males. 

The lack of difference in exercise performance observed in our study is supported by a recent literature review [12], which concluded that wearing a face mask had no impact on exercise performance. This review assessed nine studies involving a variety of populations (healthy and clinical) and exercise modalities (maximal, submaximal, aerobic and resistance exercise) when wearing cloth, surgical, or N95 masks. However, none of the reviewed studies assessed the impact of face masks on exercise performance in children and, to the authors’ knowledge, no such research has been completed until the current study. Therefore, the results of the current study are novel in that this is the first study to assess the impact of wearing a face mask on exercise performance in children. 

A lack of effect of wearing a face mask on heart rate or arterial oxygen saturation is similar to the findings of Goh et al. [14], who found no difference in heart rate or oxygen saturation in children aged 7–14 years old engaging in mild exercise while wearing a face mask (i.e., brisk treadmill walking). Similar results have been observed in adults, with the review by Shaw et al. [12] reporting no difference in heart rate or arterial oxygen saturation when wearing surgical masks. Wearing a face mask most likely leads to an increase in respiratory dead space volume which increases the fraction of inspired carbon dioxide and partial pressure of carbon dioxide in blood, potentially leading to an increased drive for ventilation [28]. This does not, however, appear to affect respiratory muscle fatigue, rating of perceived exertion, or sprint exercise performance in adults [28].

Females had greater ratings of perceived exertion for the second half of the simulated hockey period (i.e., shifts 5–7) when wearing the mask compared to the sham mask, while no difference was observed at any time point in males. While no other research has been conducted in children wearing face masks on ratings of perceived exertion to make a comparison, the results in adults are mixed, with some suggesting increased ratings of perceived exertion while exercising with a mask [16,29] and others reporting no difference [15,30,31,32]. However, after reviewing the literature, Shaw et al. [12] found no impact of surgical masks on ratings of perceived exertion when studies with a high risk of bias were excluded from analyses. When considering only submaximal exercise, surgical masks increased perceived exertion, but this effect was not present when only considering maximal exercise [12]. The sex difference in perceived exertion is contrary to Shaw et al. [15], who found no differences between adult males and females exercising while wearing face masks.

A limitation of our study is that we did not assess wearing a face mask during actual hockey games. The instrumentation required (i.e., pulse oximetry, near infrared spectroscopy) did not permit us to do physiological measurements outside of a controlled lab environment. Our skating test was limited to one evaluator and one player at a time due to COVID-19 restrictions in our province at the time of the study. Although COVID-19 transmission is prevalent during hockey [2,3] our study did not determine whether wearing face masks during hockey is effective for preventing viral transmission. There is some evidence that other COVID-19 protocols aside from masking on the ice (i.e., masking off the ice, having players arrive at the rink dressed and closing dressing rooms, restricting arena access to a limited number of masked spectators) may also be effective for preventing COVID-19 transmission [33].

## 5. Conclusions

In summary, our study found no impacts of wearing a face mask on exercise performance, heart rate, or arterial oxygen saturation in youth. Muscle oxygenation was decreased in the masked condition during the submaximal shifts (shifts 1–6) in males with no difference during the maximal seventh shift, while the inverse was observed in females (i.e., no difference in shifts 1–6 but a decrease in shift 7). However, these changes were small and likely not clinically significant. Ratings of perceived exertion were higher in females in the second half of the simulated hockey period (i.e., shifts 5–7), but not in males. Face masks can be worn by youth hockey players without any negative consequences on performance and doing so might be a practical strategy to limit the spread of infectious respiratory droplets during a game of ice hockey, especially when vaccination protocols are not available to children.

## Figures and Tables

**Table 1:** Work rate during simulated hockey period in watts.

	Shifts 1–6 Interval 1 & 3 (20 s at 40% Peak Power)	Shifts 1–6 Interval 2 (10 s at 95% Peak Power)	Shift 7 Peak Power (2 × 20 s Wingate)	Shift 7 Average Power (2 × 20 s Wingate)
**Males (mask)**	154 ± 50 W	367 ± 120 W	354 ± 101 W	275 ± 78 W
**Males (sham)**	154 ± 50 W	367 ± 120 W	331 ± 101 W	264 ± 91 W
**Females (mask)**	137 ± 43 W	326 ± 102 W	294 ± 101 W	232 ± 78 W
**Females (sham)**	137 ± 43 W	326 ± 102 W	291 ± 100 W	224 ± 92 W

## Data Availability

The data presented in this study are available on request from the corresponding author.
